# Supplementary material for: Foundational Movement Skills and Play Behaviors during Recess among Preschool Children: A Compositional Analysis
Source: Children (Basel). 2021 Jun 24;8(7):543. doi: 10.3390/children8070543 (PMC8306790; doi:10.3390/children8070543)
Supplement: Supplementary file 1 [file children-08-00543-s001.zip › children-1263558-supplementary.pdf]

children MDPI

Supplementary file. Compositional variation matrices of time spent in SOCARP variables

Table S1. Table reporting variation matrices of time spent in different activity levels.

	Lying	Sitting	Standing	Walking	Very Active
Lying	0.000	3.146	2.269	1.865	2.874
Sitting	3.146	0.000	2.966	3.405	4.624
Standing	2.269	2.966	0.000	1.526	2.913
Walking	1.865	3.405	1.526	0.000	1.686
Very Active	2.874	4.624	2.913	1.686	0.000

Note. A value close to zero implies that the two parts involved in the ratio (arranged by the rows and columns in the matrix) are highly proportional.

Table S2. Table reporting variation matrices of time spent in different group sizes.

	Alone	Small	Medium	Large
Alone	0.000	3.679	5.958	3.033
Small	3.679	0.000	3.600	2.299
Medium	5.958	3.600	0.000	2.705
Large	3.033	2.299	2.705	0.000

Note. A value close to zero implies that the two parts involved in the ratio (arranged by the rows and columns in the matrix) are highly proportional.

Table S3. Table reporting variation matrices of time spent in different activity types.

	AE	AW	Sedentary Behaviour	Inactive Play	Locomotion
AE	0.000	8.501	7.805	6.663	7.143
AW	8.501	0.000	5.454	6.474	4.017
Sedentary Behaviour	7.805	5.454	0.000	5.451	3.207
Inactive Play	6.663	6.474	5.451	0.000	5.270
Locomotion	7.143	4.017	3.207	5.270	0.000

Notes. AE: Active Games with Equipment; AW: Active Games without Equipment (%). A value close to zero implies that the two parts involved in the ratio (arranged by the rows and columns in the matrix) are highly proportional.
